# Hydrogel-Delivered Recombinant Fibronectin DK1 Promotes Diabetic Wound Healing by Boosting Cellular Responses

**DOI:** 10.3390/biom16040525

**Published:** 2026-04-01

**Authors:** Shiwen Chen, Qiwei Liu, Houan Zheng, Yiting Wang, Sheng Xiong

**Affiliations:** 1 Institute of Biomedicine & Department of Cell Biology, College of Life Science and Technology, Jinan University, Guangzhou 510632, China; 2 Guangdong Provincial Key Laboratory of Bioengineering Medicine, National Engineering Research Center of Genetic Medicine, Jinan University, Guangzhou 510632, China; 3 Guangzhou Cheer-Derm Biotech Co., Ltd., Guangzhou 510530, China

**Keywords:** recombinant fibronectin, DK1, diabetes wound healing, hydrogel

## Abstract

Diabetic wounds remain a major clinical challenge due to persistent inflammation, impaired angiogenesis, and delayed tissue regeneration. Fibronectin plays a crucial role in regulating cellular responses during wound repair, yet the therapeutic potential of recombinant fibronectin functional domains remains insufficiently explored. In this study, three recombinant fibronectin variants (D89, D8910, and DK1) were successfully designed, expressed, and purified. Among them, DK1 exhibited superior biological activity, significantly enhancing cell proliferation, adhesion, and migration in vitro. To improve its therapeutic efficacy, recombinant DK1 was incorporated into a biocompatible hydrogel delivery system. The DK1-loaded hydrogel demonstrated excellent cytocompatibility, antioxidant, and antibacterial properties. In a diabetic mouse wound model, hydrogel-mediated delivery of DK1 significantly accelerated wound closure. Histological and immunohistochemical analyses further revealed that DK1 promoted wound repair by enhancing re-epithelialization, improving collagen deposition and remodeling, reducing inflammatory responses, and stimulating angiogenesis. These findings demonstrate that recombinant fibronectin DK1 enhances cellular responses and effectively promotes diabetic wound healing, highlighting its potential as a promising biomolecular therapeutic strategy for chronic wound treatment.

## 1. Introduction

Diabetes has emerged as a significant global public health concern, with the prevalence of type 2 diabetes consistently increasing. Retinopathy, nephropathy, neuropathy, and diabetic foot ulcers (DFUs) are frequently observed complications of diabetes [1,2,3,4]. Among these, diabetic foot ulcers represent a prevalent and serious complication for individuals with diabetes, substantially diminishing their quality of life and potentially necessitating amputations. The formation of diabetic foot ulcers is intricately linked to several factors, including hyperglycemia, chronic inflammation, microcirculatory disturbances, and immune dysfunction, all of which substantially influence the wound healing process [5,6]. Studies have shown that during diabetic wound healing, macrophages exhibit a persistent pro-inflammatory phenotype, expressing high levels of pro-inflammatory molecules including IL-1β, TNF-α, and MMP-9 [7]. Hyperglycemia may induce this pro-inflammatory state, thereby exacerbating oxidative and inflammatory stress via the generation of reactive oxygen species (ROS) and tumor necrosis factor-alpha (TNF-α) [8]. The dysregulated inflammatory and oxidative processes are critical factors contributing to impaired diabetic wound healing. Despite the current treatment options, including pharmacological interventions and surgical treatments, treating diabetic wounds remains challenging, as conventional approaches (such as debridement, offloading, infection control, and growth factor application) often fail owing to the complex pathophysiology involving persistent inflammation, impaired angiogenesis, and a protease-rich milieu that degrades therapeutic proteins.

Currently, several extracellular matrix (ECM) proteins have been employed to facilitate improved diabetic wound healing. These include collagen, elastin, fibrin, fibronectin, and laminin. These proteins can bind and regulate the availability of growth factors, serving as growth factor storage systems, providing structural support, and promoting cell survival, differentiation, adhesion, and proliferation [9].

Fibronectin is a glycoprotein widely distributed in the body and plays a key role in cell adhesion, migration, and proliferation as an important component of the ECM [10]. In recent years, fibronectin has been reported to participate in the healing processes of various tissues and wounds [11,12,13,14,15]. Wound healing is a process that occurs in three main stages: inflammation, proliferation, and remodeling [16,17,18]. During the inflammatory phase, fibronectin helps stop bleeding and form clots by interacting with platelets and other cells. This provides a supportive matrix for the wound to heal [19,20,21]. In the proliferative phase, ECM-type fibronectin binds to integrins, which promoting the migration and proliferation of fibroblasts and keratinocytes, thus accelerating wound healing [22]. Furthermore, fibronectin also regulates ECM remodeling during the remodeling stage by interacting with molecules like heparin and collagen [23,24,25,26]. However, while fibronectin plays a role in wound recovery, timely clearance and degradation of fibronectin in wounds are prerequisites for tissue recovery [27]. Proteinases released early during the inflammatory response hydrolyze fibronectin and produce fragments that alter monocyte phenotypes and migration behavior, thus contributing to wound healing [28]. In chronic wounds, the hydrolytic degradation of fibronectin can, however, hinder the healing process.

Due to the susceptibility of fibronectin to degradation in the chronic wound environment, an appropriate delivery system is necessary to preserve its biological activity. Hydrogels are polymer materials with high water content and diverse physical properties. They can be designed to resemble the extracellular environment of body tissues, making them suitable for medical implants, biosensors, and drug delivery carriers. Due to their excellent biocompatibility and controllable degradability, hydrogels are widely used in wound healing [29,30]. For chronic wound treatment, hydrogels not only provide a suitable moist environment to promote wound healing [31,32], but also act as drug delivery systems to deliver bioactive molecules such as growth factors, anti-inflammatory drugs, and antioxidants to the wound site [33]. Some studies have combined hydrogels with bioactive molecules like collagen, which can enhance skin thickness, density, and flexibility in the wound area by promoting collagen synthesis, or accelerate wound healing by increasing the expression of genes such as VEGF, TGF-β1, and IL-1β [34,35,36]. Notably, recent studies have demonstrated that the transient delivery of engineered VEGF and PDGF-BB proteins in fibronectin hydrogels promotes strong angiogenesis and arteriogenesis in the skin of diabetic mice [37]. This suggests the potential of fibronectin hydrogels in treating diabetic wound healing. Therefore, the objective of this study is to construct and express fibronectin fragments with different domains, namely D89, D8910, and DK1, and compare their biological activity in terms of cell adhesion, proliferation, and migration. By selecting the most biologically active domain, DK1, we aim to combine it with hydrogels while coupling Zn^2+^, which has antimicrobial properties, and EGCG, which has antioxidant and anti-inflammatory effects, to the hydrogel. The ultimate goal is to develop a functionalized recombinant fibronectin DK1 hydrogel aimed at wound healing, particularly for diabetic wound repair.

## 2. Materials and Methods

### 2.1. Construction and Verification of Recombinant Vectors

Fibronectin consists of multiple functional domains, each of which has its specific function [38,39,40]. In this study, we designed and constructed three recombinant fibronectins: D89 (containing domains 8 and 9), D8910 (containing domains 8–10), and DK1 (which fuses the IIICS domain onto domains 8–10). The nucleotide sequences of each fibronectin domain were retrieved from the GeneBank database. These gene fragments were synthesized by Suzhou Hongxun Biotechnology Co., Ltd. (Suzhou, China). The synthesized gene fragments were then ligated into the pET-28a(+) vector using restriction enzymes (NdeI and BamHI). The constructed vectors were named pET-28a-D89, pET-28a-D8910, and pET-28a-DK1. The recombinant plasmids were transformed into *E. coli* DH5α competent cells by heat shock, and after antibiotic selection, single colonies were picked and cultured to obtain the recombinant plasmids for subsequent experiments.

To verify the correctness of the vector construction, positive clones were selected from the transformed *E. coli* colonies, and plasmids were extracted for PCR amplification. PCR amplification was performed using the primers listed in Table 1, specific to the D89, D8910, and DK1 fragments. Electrophoresis was used to analyze the PCR products to confirm whether the target gene fragments were correctly inserted into the plasmid. A positive result was confirmed when the size of the PCR products matched the expected fragment size. After successful verification, the recombinant plasmids were used for protein expression and purification experiments.

### 2.2. Expression and Purification of Recombinant Proteins D89, D8910, and DK1

The constructed pET-28a-D89, pET-28a-D8910, and pET-28a-DK1 recombinant plasmids were transformed into *Escherichia coli* BL21(DE3) expression strains. After screening for positive clones, initial cultivation was carried out in LB/Kana medium. When the OD600 reached 0.6–0.8 at 37 °C, IPTG at different concentrations (0.1–1.0 mM) was added to induce protein expression. The induction temperature was set at 37 °C for 6 h. After induction, the bacterial culture was sonicated and the soluble supernatant was collected for protein purification. To improve protein expression and solubility, expression conditions were optimized. Protein expression was induced with different IPTG concentrations (0.1 mM, 0.25 mM, 0.5 mM, 1 mM) and at different temperatures (16 °C, 25 °C, and 37 °C). The impact of these factors on protein expression was evaluated. To select the optimal induction time, samples were collected at 1, 2, 3, 4, 5, and 6 h post-induction to assess protein expression. The soluble and insoluble fractions were analyzed by SDS-PAGE to determine the optimal expression conditions. To obtain high-purity protein, Ni-NTA affinity chromatography was used to purify the D89, D8910, and DK1 proteins. First, the bacterial pellets were sonicated to obtain the soluble supernatant. After centrifugation to remove cell debris, the supernatant was subjected to Ni-NTA column affinity chromatography. Unbound proteins were washed with 1× PBS pH = 7.4 containing 20 mM imidazole, and recombinant proteins were eluted with a gradient of imidazole (50, 100, 300, 500 mM) in 1× PBS pH = 7.4. Eluted fractions were analyzed by SDS-PAGE and Western blot. After confirming that the purity reached over 90%, the protein solution was used for subsequent cellular experiments.

After Ni-NTA affinity purification, the recombinant protein was subjected to ultrafiltration concentration to remove residual endotoxins. The endotoxin level in the final protein sample was quantified using the gel limit test according to the Pharmacopoeia of the People’s Republic of China (2020 Edition, Part IV, General Chapter 1143, Method 1). As shown in Table S1, the bacterial endotoxin concentration was <0.5 EU/mL, which is below the accepted thresholds for cell culture and animal experiments, confirming that the protein was suitable for subsequent in vitro and in vivo studies.

### 2.3. SDS-PAGE and Western Blot

After sonication and obtaining the supernatant, the proteins were analyzed by SDS-PAGE. Protein samples were mixed with 5× Loading Buffer and incubated at 100 °C for 10 min. A 12% polyacrylamide gel was prepared, and electrophoresis was performed in a tank at 80 V for the stacking gel and 120 V for the separating gel. After electrophoresis, the gel was stained with Coomassie Brilliant Blue and destained. The gel image was recorded using a gel imaging system. The molecular weight and purity of the protein were determined by comparing the protein’s migration with molecular weight markers.

After SDS-PAGE, the proteins were transferred to a PVDF membrane using a transfer method. The membrane was blocked with 5% skimmed milk for 1 h at room temperature. Then, the membrane was incubated overnight with a rabbit anti-His-tag primary antibody (1:5000 dilution). After washing the membrane 4 times with TBST (5–10 min each), the membrane was incubated with a HRP-conjugated goat anti-rabbit secondary antibody (1:5000 dilution) for 1–2 h. The membrane was washed 4 times with TBST. ECL reagent was used for detection, and chemiluminescent signals were captured with a gel imaging system to analyze the expression of the target protein.

### 2.4. In Vitro Biological Activity Evaluation

#### 2.4.1. Cell Viability

To assess cell viability, a CCK-8 assay was performed. After cell culture, cells were counted and plated at 3 × 10^3^ cells/well in a 96-well plate for 24 h. After serum starvation with DMEM for 2 h, the medium was replaced with DMEM containing 0.4% FBS and different concentrations of fibronectin. The cells were then incubated for an additional 24 h. After incubation, the medium was aspirated, and 10 μL of CCK-8 reagent was added to each well for 1.5 h. The absorbance at 450 nm and 630 nm was measured with a microplate reader, and the cell proliferation rate was calculated to evaluate the effect of fibronectin on cell proliferation.

Cell viability (%) = (OD__sample_ − OD__blank_)/(OD__control_ − OD__blank_) × 100

#### 2.4.2. Cell Adhesion

To evaluate cell adhesion, a CCK-8 assay was performed. Recombinant fibronectin at different concentrations was added to the wells and incubated overnight at 4 °C. After the incubation, the fibronectin solution was removed, and cells were plated at 1 × 10^4^ cells/well in a 96-well plate. After 1.5 h of incubation, CCK-8 reagent was added, and cells were incubated at 37 °C for 2 h. Absorbance was measured at 450 nm and 630 nm. The adhesion rate was calculated.

Cell adhesion rate (%) = (OD__sample_ − OD__blank)_/(OD__control_ − OD__blank)_ × 100

To further assess cell adhesion, a crystal violet assay was performed. Recombinant fibronectin at different concentrations was added to the wells and incubated overnight at 4 °C. After the incubation, the fibronectin solution was removed, and cells were plated at 1 × 10^4^ cells/well. After 1.5 h, 100 μL of 4% paraformaldehyde was added for fixation for 30 min, followed by three washes with 1× PBS. Cells were stained with 0.1% crystal violet solution for 30 min. After staining, images were captured with a fluorescence microscope (Nikon, Eclipse Ti-E, Tokyo, Japan). Eight random fields per well were captured by a blinded operator, and the number of cells was counted using Image-Pro Plus 6.0 software to calculate the adhesion rate.

#### 2.4.3. Cell Migration

To assess cell migration, a scratch wound assay was performed. The scratch test was repeated in 3 biological replicates. For each replicate, 3 random scratch areas per well were chosen (n = 9 per group). Cells were seeded at a density of 1 × 10^6^ cells/mL in a 6-well plate. A 10 μL pipette tip was used to create a scratch wound at 0 h. The control group was cultured in DMEM with 1% FBS, and the experimental group was cultured with different concentrations of FN in DMEM with 1% FBS. After 24 h of incubation with the samples, cell migration was observed and imaged with an inverted fluorescence microscope (Olympus, IX53, Tokyo, Japan). Migration area at 24 h was measured using Image-Pro Plus 6.0 software and compared to the initial wound area at 0 h to calculate the wound healing rate.

### 2.5. Hydrogel Preparation and Compatibility Evaluation

#### 2.5.1. Hydrogel Preparation and Physical Characterization

The hydrogel system was constructed using phenylboronic acid-grafted hyaluronic acid (HA-PBA, 15 mg/mL), oxidized dextran (ODex, 50 mg/mL), and gelatin (Type A, porcine skin, Sigma-Aldrich (Saint Louis, MO, USA), 50 mg/mL). All components were dissolved in PBS (pH 7.4). HA-PBA was prepared as Solution A; ODex, gelatin, Zn-EGCG complex, and DK1 were prepared as Solution B. The Zn-EGCG complex was pre-formed by dissolving ZnSO_4_·7H_2_O and EGCG in PBS at a 1:1 mass ratio (final concentration of 200 μg/mL each). DK1 was added at a concentration of 750 μg/mL (optimized based on Section 3.5). The crosslinking agents employed include 2-aminophenylboronic acid, 4-(4,6-dimethoxy-1,3,5-triazin-2-yl)-4-methylmorpholinium chloride, and sodium periodate. Upon mixing Solutions A and B, gelation occurred immediately through dual dynamic covalent crosslinking: boronate ester bonds between HA-PBA and ODex, and Schiff base bonds between aldehyde groups of ODex and amino groups of gelatin and DK1. Therefore, DK1 was incorporated through reversible Schiff base formation, physical entrapment within the dual crosslinked network, and non-covalent interactions, enabling sustained release while preserving its bioactivity. The blank hydrogel (HA-PBA/ODex/gelatin) was rheologically characterized using a rheometer (Kinexus Pro+, Malvern, UK), exhibiting a storage modulus (G’) of 754.6 Pa (Figure S1A); it reached swelling equilibrium in PBS at 37 °C over 72 h, with a swelling ratio of 139 ± 3.3% (Figure S1B).

#### 2.5.2. Cell Viability

To evaluate the cell viability of the DK1 hydrogel, a CCK-8 assay was performed. After cell culture and counting, cells were plated at 1 × 10^4^ cells/well (100 μL per well) in a 96-well plate and incubated overnight for 24 h. The hydrogel was placed in a 50 mL centrifuge tube and soaked in serum-free DMEM medium (1:10 mass-to-volume ratio) at 37 °C for 24 h with shaking. The extract was filtered using a 0.22 μm filter and prepared into a 10% FBS hydrogel extract. Cells were serum-starved for 2 h, and then the medium was replaced with DMEM containing 10% FBS and different hydrogel extracts. After 24 h of incubation, 10 μL of CCK-8 reagent was added to the wells and incubated for 1.5 h. Absorbance was measured at 450 nm and 630 nm, and the cell proliferation rate was calculated.

Cell viability (%) = (OD__sample_ − OD__blank_)/(OD__control_ − OD__blank_) × 100

#### 2.5.3. Cell Migration

To assess the cell migration ability of the DK1 hydrogel, a scratch wound assay was performed. The scratch test was repeated in 3 biological replicates. For each replicate, 3 random scratch areas were selected per well (n = 9 per group). Cells at a density of 1 × 10^6^ cells/mL were seeded into 6-well plates. A 10 μL pipette tip was used to create a scratch wound at 0 h. The control group was cultured in DMEM with 1% FBS, while the experimental group was cultured with DMEM containing 1% FBS and different hydrogel extracts. After 24 h of incubation with the samples, cell migration was observed and imaged using an inverted fluorescence microscope (Olympus, IX53, Tokyo, Japan). Migration areas at 24 h were measured using Image-Pro Plus 6.0 software and compared to the initial wound area at 0 h to calculate the wound healing rate.

### 2.6. Antioxidant and Antibacterial Performance of the Hydrogel

#### 2.6.1. Antioxidant Performance Evaluation

To evaluate the antioxidant performance of the DK1 hydrogel, a CCK-8 assay was performed. After cell culture and counting, cells were plated at 1 × 10^4^ cells/well (100 μL per well) in a 96-well plate and incubated overnight for 24 h. After incubation, cells were treated with 0.1 mM H_2_O_2_ for 1.5 h to simulate oxidative damage, and then different hydrogel extracts were added to the cells in DMEM with 1% FBS. After 24 h of incubation, 10 μL of CCK-8 reagent was added to each well and incubated for 1.5 h. The absorbance at 450 nm and 630 nm was measured with a microplate reader. Cell survival rates were calculated to assess the antioxidant performance of the DK1 hydrogel.

#### 2.6.2. Antibacterial Performance Evaluation

To evaluate the antibacterial performance of the DK1 hydrogel, 200 μL of the hydrogel was added to each well of a 48-well plate. Subsequently, 100 μL of bacterial suspension (1 × 10^6^ cfu/mL) was added to the surface of each hydrogel. Each well also received 1 mL of sterile LB medium. For the control group, 100 μL of bacterial suspension was added to 1 mL of sterile LB medium. The 48-well plate was incubated at 37 °C for 4–6 h. After incubation, the suspensions were transferred to sterile tubes. The suspensions were diluted, and 100 μL of the diluted suspension was spread on LB agar plates without antibiotics. The plates were incubated overnight at 37 °C, and the number of bacterial colonies was counted the next day. Each group was repeated three times to assess the antibacterial capacity.

Antibacterial activity = (Number of colonies in the control group − Number of colonies in the hydrogel group)/ Number of colonies in the control group

### 2.7. In Vivo Wound Healing Evaluation

#### 2.7.1. Establishment of the Diabetic Mouse Wound Model

All animal experiments were approved by the Experimental Animal Ethics Committee of Jinan University (IACUC-20250801-15). Male C57BL/6J mice (190–210 g, 6 weeks old) were purchased from Beijing Vital River Experimental Animal Technology Co., Ltd. (Beijing, China). To construct the diabetic mouse model, STZ (streptozotocin) was used to damage the pancreatic islet cells of the mice, inducing diabetes. A low-dose model was created by administering STZ at 60 mg/kg via intraperitoneal injection once a day for five consecutive days. After three weeks, when the blood glucose level stabilized above 16.7 mmol/L, along with the typical symptoms of “polyuria, polydipsia, polyphagia, and weight loss,” the model was considered successfully established. The mice were then divided into six groups: negative control group (saline), HG group, HG + Zn^2+^-EGCG group, HG + 750 DK1 group, HG + Zn^2+^-EGCG + 750DK1 group, and positive control group (Aquacel AG dressing) and contained 3 mice in each group, with a total of 18 mice used in the in vivo wound healing experiment. To reduce potential confounding factors, all animals were maintained under identical housing conditions, and treatments were administered in a balanced manner across experimental groups. Mice were anesthetized with sodium pentobarbital, their backs were shaved and depilatory cream was applied, followed by disinfection with alcohol. A full-thickness wound was created using an 8 mm punch on the back of the mice, down to the fascia layer, serving as the wound repair model. All surgeries were conducted under sterile conditions.

#### 2.7.2. Wound Treatment and Sample Collection in Mice

On the day following the wound creation, saline was applied to the negative control group, silver ion dressing (Aquacel AG, Purchased from ConvaTec (China) Medical Supplies Co., Ltd., Shanghai, China) was applied to the positive control group, and different hydrogel formulations were applied to the experimental groups. Wound healing was evaluated starting from day 0, and at days 3, 7, 10, and 12, wound areas were recorded and photographed. Wound areas were measured by tracing the wound boundary on graph paper, and random mouse euthanasia was performed for sample collection at each time point for subsequent histological analysis.

Wound healing rate = (Initial wound area − Wound area at the target day)/Initial wound area.

Randomization and blinding: Animal group allocation was concealed during surgery and outcome assessment. Mice were randomly assigned to treatment groups using a computer-generated random number table. Wound area measurements and histological analyses were performed by investigators blinded to treatment assignments.

### 2.8. Immunohistochemical Analysis

To evaluate epidermal regeneration and inflammation in the wound area, tissue samples collected on days 3, 7, 10, and 12 were fixed in 4% paraformaldehyde for 1 h. The samples were then embedded in paraffin and sectioned into 4 μm-thick slices. Hematoxylin and eosin (H&E) staining, as well as Masson’s trichrome staining, were used for histological analysis. All slices were analyzed, and images were captured using a microscope (IX53, Olympus, Tokyo, Japan). For immunofluorescence staining, regeneration of skin in the wound area was examined on days 3, 7, 10, and 12, with the expression of CD68, CD31, COL-I, COL-III, and other markers assessed to evaluate inflammation, angiogenesis, and collagen deposition during wound healing. Fluorescence signals were observed under a fluorescence microscope, and the expression of related proteins in the tissue at different time points was quantified.

### 2.9. Data Analysis

All data are expressed as means ± standard deviation (SD), based on multiple individual batches. Origin 2021 software was used for data analysis. After confirming normality (Shapiro–Wilk test) and homogeneity of variance (Levene’s test), one-way analysis of variance (ANOVA) was used for comparisons among three or more groups, followed by Tukey’s post hoc test. A *p*-value < 0.05 was considered statistically significant: (*) for *p* < 0.05, (**) for *p* < 0.01, (***) for *p* < 0.001, (****) for *p* < 0.0001.

## 3. Results

### 3.1. Construction and Verification of D89, D8910, and DK1

**Figure 1:**
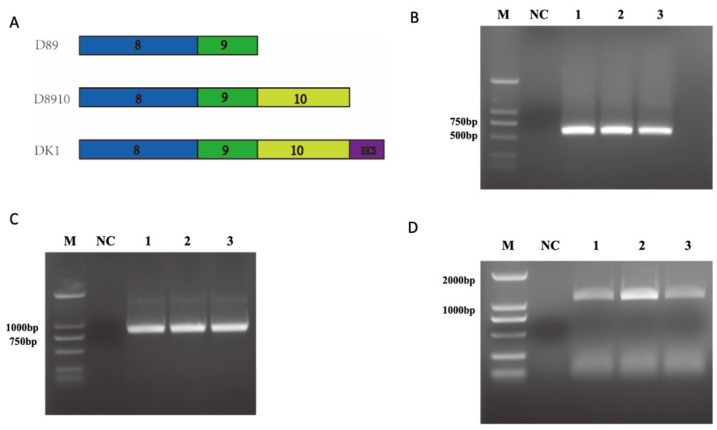
Construction and Verification of D89, D8910, and DK1. (**A**) Schematic diagram of the gene constructs for D89, D8910 and DK1. (**B**) Electrophoresis results of the PCR product for the recombinant plasmid pET-28a-D89. M: DNA maker, NC: negative control, 1–3: three randomly selected monoclonal PCR products. (**C**) Electrophoresis results of the PCR product for the recombinant plasmid pET-28a-D8910. The lettering on the image corresponds to that in (**B**). (**D**) Electrophoresis results of the PCR product for the recombinant plasmid pET-28a-DK1. The lettering on the image corresponds to that in (**B**).

Fibronectin is a large protein that contains multiple functional domains, each with its specific function [41]. To investigate the biological functions of specific fibronectin domains, this study designed and constructed three recombinant fibronectin proteins: D89 (containing domains 8 and 9), D8910 (containing domains 8–10), and DK1 (fusion of the IIICS domain with domains 8–10) (Figure 1A). The D89, D8910, and DK1 constructs were inserted into the pET-28a expression vector. Colony PCR and agarose gel electrophoresis results showed specific bands at 567 bp, 840 bp, and 1083 bp, corresponding to the theoretical lengths of the D89, D8910, and DK1 genes, respectively (Figure 1B–D). This confirmed that the recombinant plasmids pET-28a-D89, pET-28a-D8910, and pET-28a-DK1 were successfully constructed.

### 3.2. Expression and Purification of D89, D8910, and DK1

**Figure 2:**
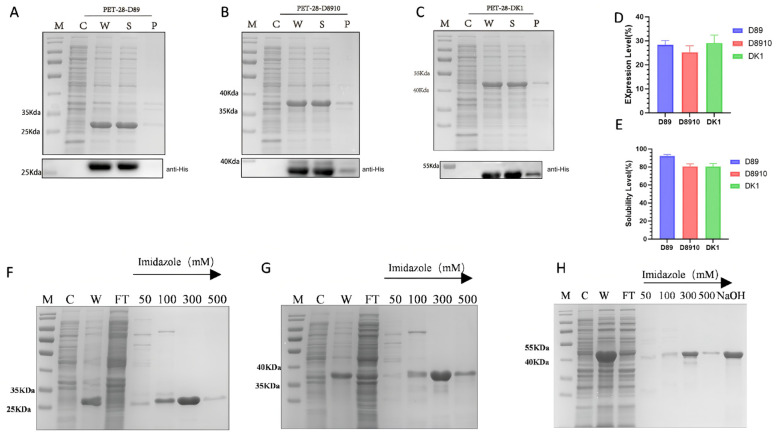
Expression and Purification of D89, D8910, and DK1. (**A**) SDS-PAGE and Western blot analysis of D89 expression. Lanes: M: Protein marker C: Uninduced negative control. W: Induced bacterial cells. S: Induction supernatant. P: induced pellet. (**B**) SDS-PAGE and Western blot analysis of D8910 expression. The lane configuration depicted in the diagram is identical to that in (**A**). (**C**) SDS-PAGE and Western blot analysis of DK1 expression. The lane configuration depicted in the diagram is identical to that in (**A**). (**D**) Analysis of Expression Levels of Three Recombinant Fibronectin Variants. (**E**) Analysis of Solubility Levels in Three Recombinant Fibronectin Variants. (**F**) SDS-PAGE analysis of D89 purified by Ni-NTA affinity chromatography. Lanes: M: Protein marker; C: Uninduced negative control. W: Induced bacterial cells. FT: Flow-through; 50/100/300/500: Elution fractions. (**G**) SDS-PAGE analysis of D8910 purified by Ni-NTA affinity chromatography. The lane configuration depicted in the diagram is identical to that in (**F**). (**H**) SDS-PAGE analysis of DK1 purified by Ni-NTA affinity chromatography. The lane configuration depicted in the diagram is identical to that in (**F**).

To verify the expression of the three recombinant fibronectin proteins, the successfully constructed pET-28a-D89, pET-28a-D8910, and pET-28a-DK1 were transformed into the BL21(DE3) *E. coli* expression host and cultured at 37 °C for 6 h. The results showed that D89, D8910, and DK1 produced specific bands at 22 kDa, 32 kDa, and 40 kDa, respectively, which matched the predicted molecular weights of the proteins, confirming the successful expression of all three recombinant fibronectins (Figure 2A–C). Further analysis of the solubility of the expressed proteins showed that the expression levels of all three proteins accounted for over 25% of the total protein (Figure 2D), and the soluble expression ratios were all over 80%, with D89 reaching the highest at 92.23% (Figure 2E). To determine the optimal expression conditions for the three recombinant strains, we tested different induction temperatures, IPTG concentrations, and induction times. The optimal conditions for all three strains were found to be 37 °C, 0.1 mM IPTG, and 6 h of induction (Figures S2–S4). To obtain high-purity proteins, Ni-NTA affinity chromatography was used to purify D89, D8910, and DK1. The results showed that D89 was purified with high purity (99.1%) using elution buffers containing 300 mM and 500 mM imidazole (Figure 2F). Similarly, D8910 and DK1 were also purified with high purity (97.04% and 92.8%, respectively) using 300 mM and 500 mM imidazole elution buffers (Figure 2G,H).

### 3.3. In Vitro Activity Evaluation of D89, D8910, and DK1

**Figure 3:**
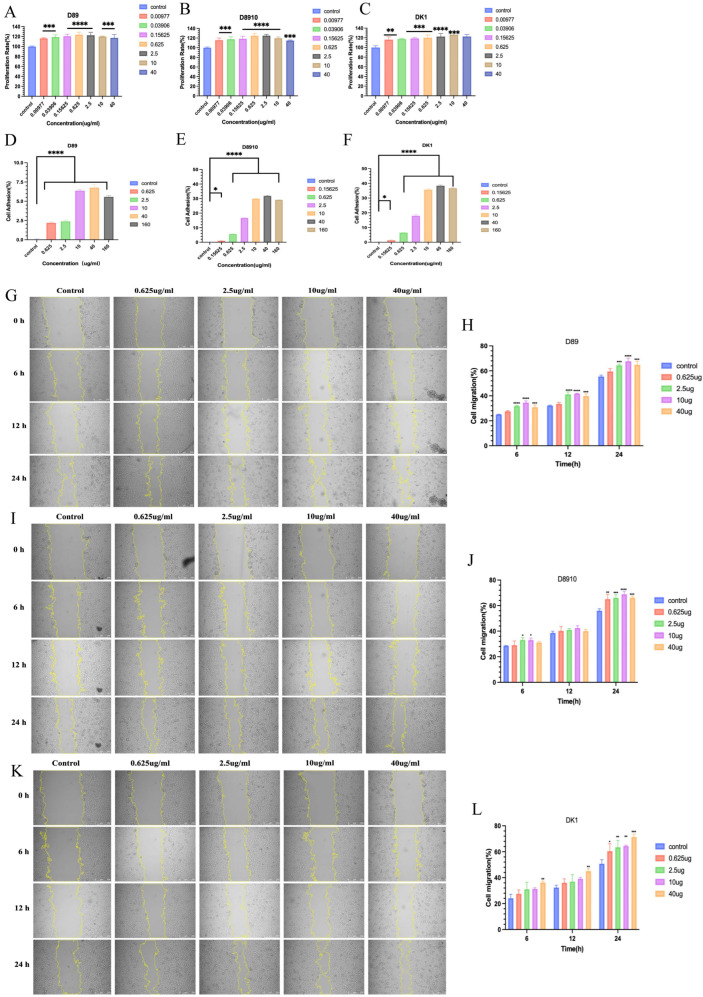
In vitro activity assessment of D89, D8910, and DK1. (**A**) Quantification of NIH/3T3 cell proliferation rates at different D89 concentrations. (**B**) Quantification of NIH/3T3 cell proliferation rates at different D8910 concentrations. (**C**) Quantification of NIH/3T3 cell proliferation rates at different DK1 concentrations. (**D**) Quantification of NIH/3T3 cell adhesion rates at different D89 concentrations. (**E**) Quantification of NIH/3T3 cell adhesion rates at different D8910 concentrations. (**F**) Quantification of NIH/3T3 cell adhesion rates at different DK1 concentrations. (**G**) Cell migration: Images of scratch-wounded monolayer HUVECs cultured in 6-well plates at 0 h, and migration images of HUVECs co-cultured with different concentrations of D89 at 6, 12, and 24 h. Scale bar = 250 μm. (**H**) Quantification of D89 effects on HUVEC migration. (**I**) Cell migration: images of scratch-wounded monolayer HUVECs cultured in a 6-well plate at 0 h, and migration of HUVECs co-cultured with different concentrations of D8910 at 6, 12, and 24 h. Scale bar = 250 μm. (**J**) Quantification of D8910 effects on HUVEC migration. (**K**) Cell migration: Images of scratch-injured monolayer HUVECs cultured in a 6-well plate at 0 h, and migration of HUVECs co-cultured with different concentrations of DK1 at 6, 12, and 24 h. Scale bar = 250 μm. (**L**) Quantification of DK1 effects on HUVEC migration. Compared to the control group, A *p*-value < 0.05 was considered statistically significant: (*) for *p* < 0.05, (**) for *p* < 0.01, (***) for *p* < 0.001, (****) for *p* < 0.0001.

The in vitro activity of D89, D8910, and DK1 was first assessed using the CCK-8 assay with NIH/3T3 fibroblasts. The results showed that all three recombinant fibronectins significantly promoted 3T3 cell proliferation, with a dose-dependent effect as protein concentration increased. Specifically, D89 achieved the highest proliferation rate (24%) at 0.625 μg/mL (Figure 3A), D8910 reached the maximum proliferation rate (25.13%) at 2.5 μg/mL (Figure 3B), and DK1 showed the highest proliferation rate (26.36%) at 10 μg/mL (Figure 3C). However, there was no significant difference in the proliferative effects of the three proteins on 3T3 cells at the same concentration (Figure S5A). We further evaluated the effects of D89, D8910, and DK1 on the adhesion of NIH/3T3 fibroblasts at different concentrations. The results showed that D89, D8910, and DK1 all had a dose-dependent effect on cell adhesion. At a concentration of 40 μg/mL, the maximum adhesion rates of D89, D8910, and DK1 were 6.77%, 31.84%, and 38.43%, respectively (Figure 3D–F), indicating that all three recombinant fibronectins promoted cell adhesion. Notably, DK1 exhibited the best adhesion-promoting effect at the same concentration compared to D89 and D8910 (Figure S5B). Additionally, we evaluated the effects of D89, D8910, and DK1 on HUVEC migration at different concentrations and time points. Scratch assay results showed that D89, D8910, and DK1 significantly promoted HUVEC migration, exhibiting a dose-dependent effect (Figure 3G,I,K). Specifically, at 24 h, D89 showed a wound healing rate of 64.5%, 67.78%, and 64.92% at concentrations of 2.5 μg/mL, 10 μg/mL, and 40 μg/mL, respectively, all significantly higher than the control group (Figure 3H). D8910 achieved wound healing rates of 65.15%, 66.11%, 68.86%, and 66.25% at concentrations of 0.625 μg/mL, 2.5 μg/mL, 10 μg/mL, and 40 μg/mL, respectively, significantly higher than the control group (Figure 3J). Notably, DK1 exhibited the best migration-promoting effect at 24 h at a concentration of 40 μg/mL, with a healing rate of 71.39% (Figure 3L). When comparing the migration capabilities of the three recombinant fibronectins, DK1 consistently performed better than D89 and D8910 at multiple time points and concentrations (Figure S6A–C).

### 3.4. DK1 Promotes Adhesion of HaCaT and HUVECs

**Figure 4:**
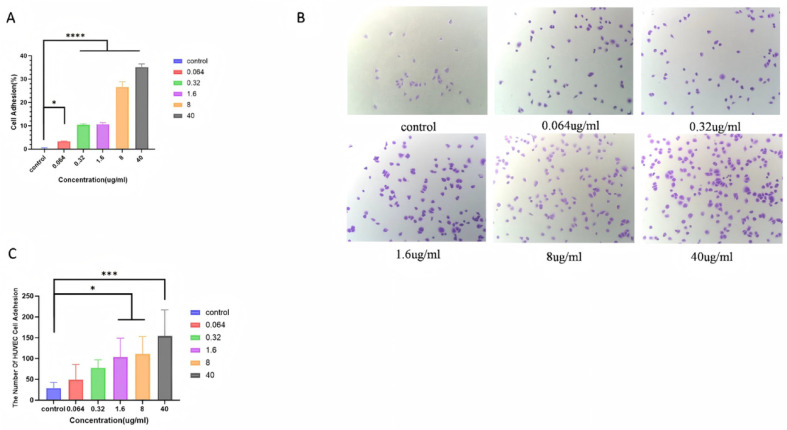
Evaluation of DK1 on Adhesion Activity of HaCaT and HUVECs. (**A**) Quantification of DK1-treated HaCaT cell adhesion rate. (**B**) Morphology of adherent cells observed via crystal violet staining after 24-h treatment of HUVECs with varying DK1 concentrations. (**C**) Quantification of DK1-treated HUVEC adhesion rate. Compared to the control group, A *p*-value < 0.05 was considered statistically significant: (*) for *p* < 0.05, (***) for *p* < 0.001, (****) for *p* < 0.0001.

Based on the superior in vitro activity of DK1, we further investigated its adhesion-promoting effect on two skin-related cell lines: HaCaT and HUVEC. First, we assessed the adhesion activity of DK1 on HaCaT cells using the CCK-8 assay. The results showed that the adhesion rate of HaCaT cells increased in a dose-dependent manner from 0.064 μg/mL to 40 μg/mL of DK1, reaching a maximum adhesion rate of 35.17% at 40 μg/mL (Figure 4A). Furthermore, we evaluated the adhesion of HUVECs to DK1 using the crystal violet staining method. The results showed that the adhesion of HUVECs also exhibited dose dependence from 0.064 μg/mL to 40 μg/mL of DK1, with the best adhesion observed at 40 μg/mL (Figure 4B,C).

### 3.5. In Vitro Biocompatibility of DK1 Hydrogel

**Figure 5:**
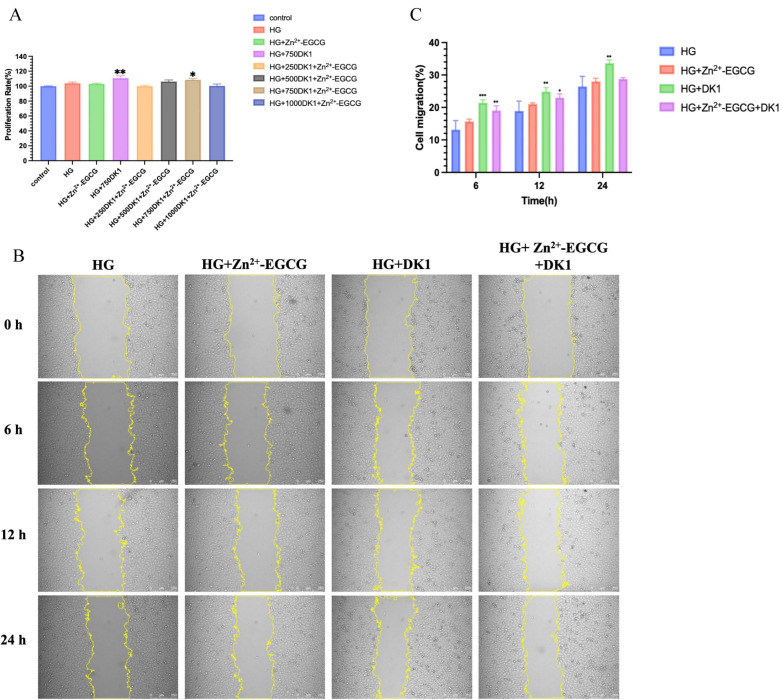
In vitro biocompatibility study of DK1 hydrogels. (**A**) Quantification of HUVEC prolifer-ation rates following coupling of DK1 at different concentrations with hydrogels containing Zn^2+^-EGCG. HG: Hydrogel; 250DK1: 250 μg/mL DK1-conjugated hydrogel; 500DK1: 500 μg/mL DK1-conjugated hydrogel; 750DK1: 750 μg/mL DK1-conjugated hydrogel; 1000DK1: 1000 μg/mL DK1-conjugated hydrogel; (**B**) Cell migration. Images of scratch-injured monolayer HUVECs cultured in 6-well plates at 0 h, and after 6, 12, and 24 h with different media (including HG, HG + Zn^2+^-EGCG, HG + DK1, and HG + Zn^2+^-EGCG +DK1) at 6, 12, and 24 h post-co-culture, with DK1 at 750 μg/mL in all cases. Scale bar = 250 μm. (**C**) Quantification of HUVEC migration rates. Compared to the control group or the HG group. A *p*-value < 0.05 was considered statistically significant: (*) for *p* < 0.05, (**) for *p* < 0.01, (***) for *p* < 0.001.

The in vitro biocompatibility of DK1 hydrogel was assessed using CCK-8 assays and cell migration tests with HUVECs. The results indicated that DK1 hydrogel did not significantly inhibit the proliferation of HUVECs. Additionally, we evaluated the coupling effect of DK1 at different concentrations with the hydrogel. Compared to the hydrogel alone, DK1 hydrogel at a concentration of 750 µg/mL showed the best cell proliferation-promoting ability (Figure 5A). Based on the CCK-8 results, the optimal coupling concentration of DK1 with the hydrogel was 750 µg/mL. The cell migration effect of the HG+ DK1 group was better than the other groups at 6 h, 12 h, and 24 h (Figure 5B). The migration rates at these time points were 21.46%, 24.9%, and 33.65%, respectively (Figure 5C).

### 3.6. Evaluation of the Antioxidant and Antibacterial Properties of DK1 Hydrogel

**Figure 6:**
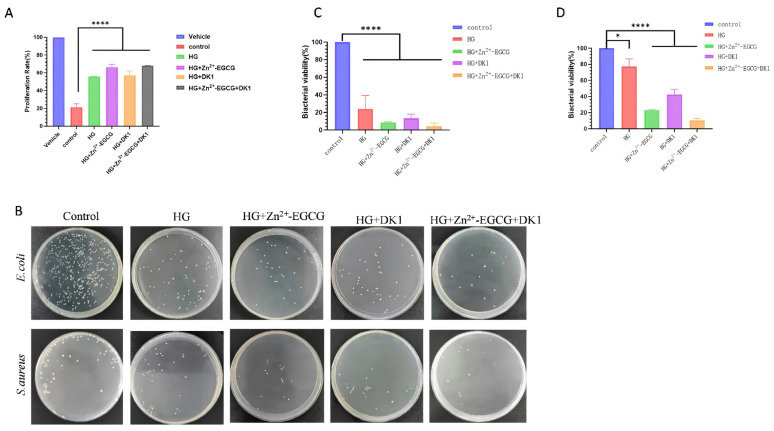
Evaluation of the Anti-inflammatory and Antibacterial Properties of DK1 Hydrogel. (**A**) Quantification of HUVEC proliferation. Includes Vehicle: negative control, control: H_2_O_2_-treated positive control, HG, HG + Zn^2+^-EGCG, HG + DK1, and HG + Zn^2+^-EGCG + DK1. (**B**) Plate spread patterns of *E. coli* and *S. aureus*, including Control, HG, HG + Zn^2+^-EGCG, HG + DK1, and HG + Zn^2+^-EGCG + DK1. (**C**) Quantification of *E. coli* viability. (**D**) Quantification of *S. aureus* viability. Compared to the control group, A *p*-value < 0.05 was considered statistically significant: (*) for *p* < 0.05, (****) for *p* < 0.0001.

The antioxidant and antibacterial properties of the hydrogel are important indicators for wound healing applications. To assess the antioxidant capability of DK1 hydrogel, we used H_2_O_2_ to induce oxidative damage in HUVECs. The results showed that the cell survival rate in the model group significantly decreased, whereas the cell survival rates in the HG, HG + Zn^2+^-EGCG, HG + DK1, and HG + Zn^2+^-EGCG + DK1 treatment groups were all significantly higher than the model group (Figure 6A). Additionally, we further evaluated the antibacterial properties of DK1 hydrogel using *E. coli* and *Staphylococcus aureus*. The results showed that DK1 hydrogel effectively inhibited the growth of both *E. coli* and *Staphylococcus aureus*, demonstrating good antibacterial activity (Figure 6B), especially in the HG + Zn^2+^-EGCG + DK1 group (Figure 6C,D). These results indicate that DK1 hydrogel not only has antioxidant properties but also possesses antibacterial function, providing additional value for wound healing applications.

### 3.7. DK1 Hydrogel Promotes Diabetic Wound Healing in Mice

**Figure 7:**
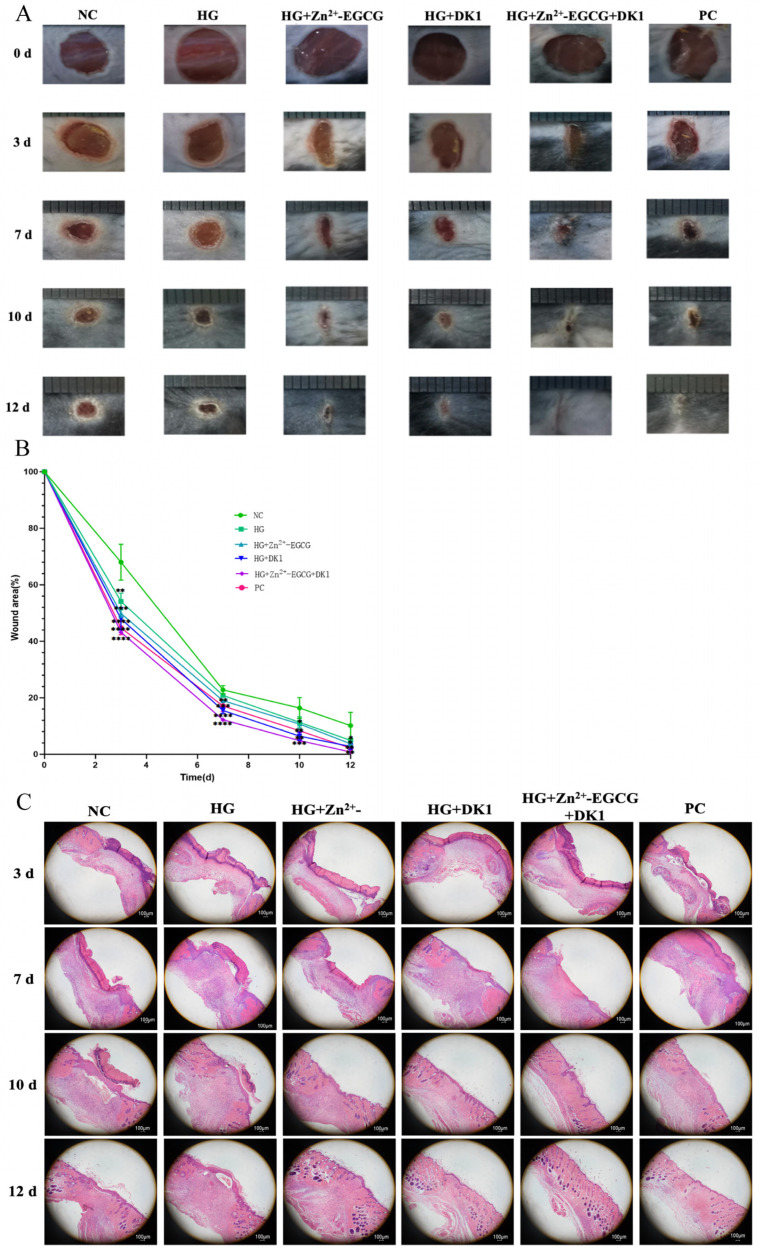
DK1 Hydrogel Promotes Wound Healing in Diabetic Mice. (**A**) Wound photographs at Days 0, 3, 7, 10, and 12. NC: saline-treated; Treatment groups: HG, HG + Zn^2+^-EGCG, HG + DK1, and HG + Zn^2+^-EGCG + DK1.PC: Aquacel AG dressing treatment. (**B**) Quantification of wound healing area. Compared to the NC group, A *p*-value < 0.05 was considered statistically significant: (*) for *p* < 0.05, (**) for *p* < 0.01, (***) for *p* < 0.001, (****) for *p* < 0.0001. (**C**) Wound HE staining at Days 0, 3, 7, 10, and 12; scale bar = 100 μm.

After successfully establishing a diabetic mouse model using STZ (streptozotocin), we treated the mice with NC (saline), HG, HG + Zn^2+^-EGCG, HG + 750 DK1, HG + Zn^2+^-EGCG + 750 DK1, and PC (aquacel ag dressing). The results showed representative images of the wound area at 0, 3, 7, 10, and 12 days post-surgery (Figure 7A). The HG + Zn^2+^-EGCG + DK1 group showed the best wound healing effects at all time points after surgery, with wound healing rates of 56.79%, 87.78%, 95.15%, and 99.19% at post-surgery days 3, 7, 10, and 12, respectively. Notably, at day 12, the HG + Zn^2+^-EGCG + DK1 group achieved a healing rate of 99.19%, significantly higher than the negative control group (89.86%) and slightly higher than the positive control group (97.83%) (Figure 7B). Histological analysis of the regenerated dermis at days 3, 7, 10, and 12 revealed that the HG + Zn^2+^-EGCG + DK1 group had significantly reduced inflammation by day 7, while other groups still exhibited substantial inflammatory infiltration. At day 10, the HG + Zn^2+^-EGCG + DK1 group exhibited the best recovery with clear regeneration of skin appendages, epidermis, and dermal thickening, while other groups showed necrosis, scabs, and inflammatory cells in the dermis. By day 12, the HG + Zn^2+^-EGCG + DK1 group’s wound area closely resembled normal skin structure, with abundant skin appendages regenerated, while the negative control group showed thickened epidermis, still visible inflammatory cells in the dermis, and no significant changes in skin appendages (Figure 7C).

### 3.8. DK1 Hydrogel Promotes Collagen Deposition and Remodeling in Wounds

**Figure 8:**
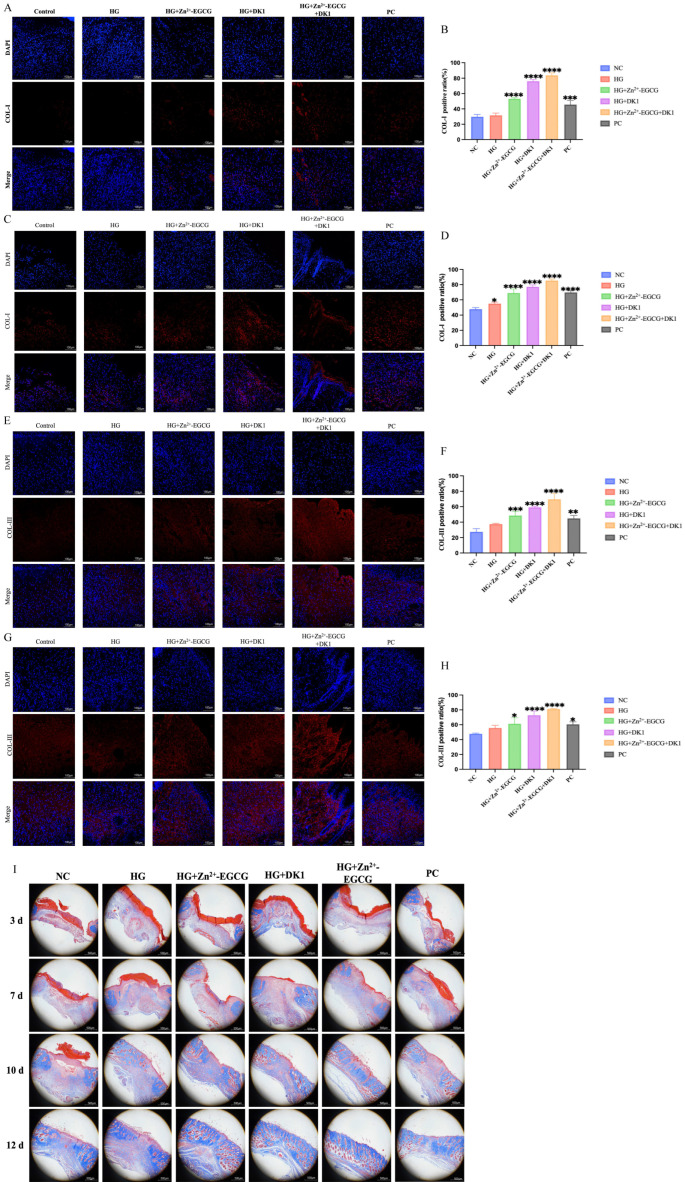
Evaluation of DK1 Hydrogel on Wound Collagen Deposition and Remodeling. (**A**) Immunofluorescence staining images of COL-I expression at the wound site on day 7 post-surgery, scale bar 100 μm. (**B**) Quantification of COL-I expression at the wound site on day 7 post-surgery. (**C**) Immunofluorescence staining images of COL-I expression at the wound site on day 10 post-surgery. (**D**) Quantification of COL-I expression at the wound site on day 10 post-surgery. (**E**) Immunofluorescence staining image of COL-III expression at the wound site on day 7 post-surgery. (**F**) Quantification of COL-III expression at the wound site on day 7 post-surgery. (**G**) Immunofluorescence staining image of COL-III expression at the wound site on day 10 post-surgery. (**H**) Quantification of COL-III expression at the wound site on day 10 post-surgery. (**I**) Wound Masson staining at Days 0, 3, 7, 10, and 12; blue indicates collagen fibers, scale bar = 500 μm. Compared to the NC group, A *p*-value < 0.05 was considered statistically significant: (*) for *p* < 0.05, (**) for *p* < 0.01, (***) for *p* < 0.001, (****) for *p* < 0.0001.

Appropriate collagen deposition and remodeling can enhance tissue tensile strength, leading to better healing outcomes. COL-I and COL-III are the major extracellular matrix (ECM) components in the dermis, and their formation is indispensable for wound healing. Therefore, we performed immunofluorescence staining for COL-I and COL-III on wound tissues at days 7 and 10. As the healing time progressed, COL-I and COL-III deposition increased in all wound sites, with the HG + Zn^2+^-EGCG + DK1 group showing the best deposition effects (Figure 8A–H). Additionally, we assessed collagen deposition in the healing site using Masson staining. On day 7, the HG + DK1 and HG + Zn^2+^-EGCG + DK1 groups displayed better collagen deposition and arrangement with reduced inflammation. From days 10 to 12, the HG + Zn^2+^-EGCG + DK1 group continued to show superior repair advantages, with collagen structures resembling normal skin and accelerated regeneration of skin appendages such as hair follicles. This effect was overall better than in other groups (Figure 8I).

### 3.9. DK1 Hydrogel Reduces Wound Inflammation and Promotes Angiogenesis

**Figure 9:**
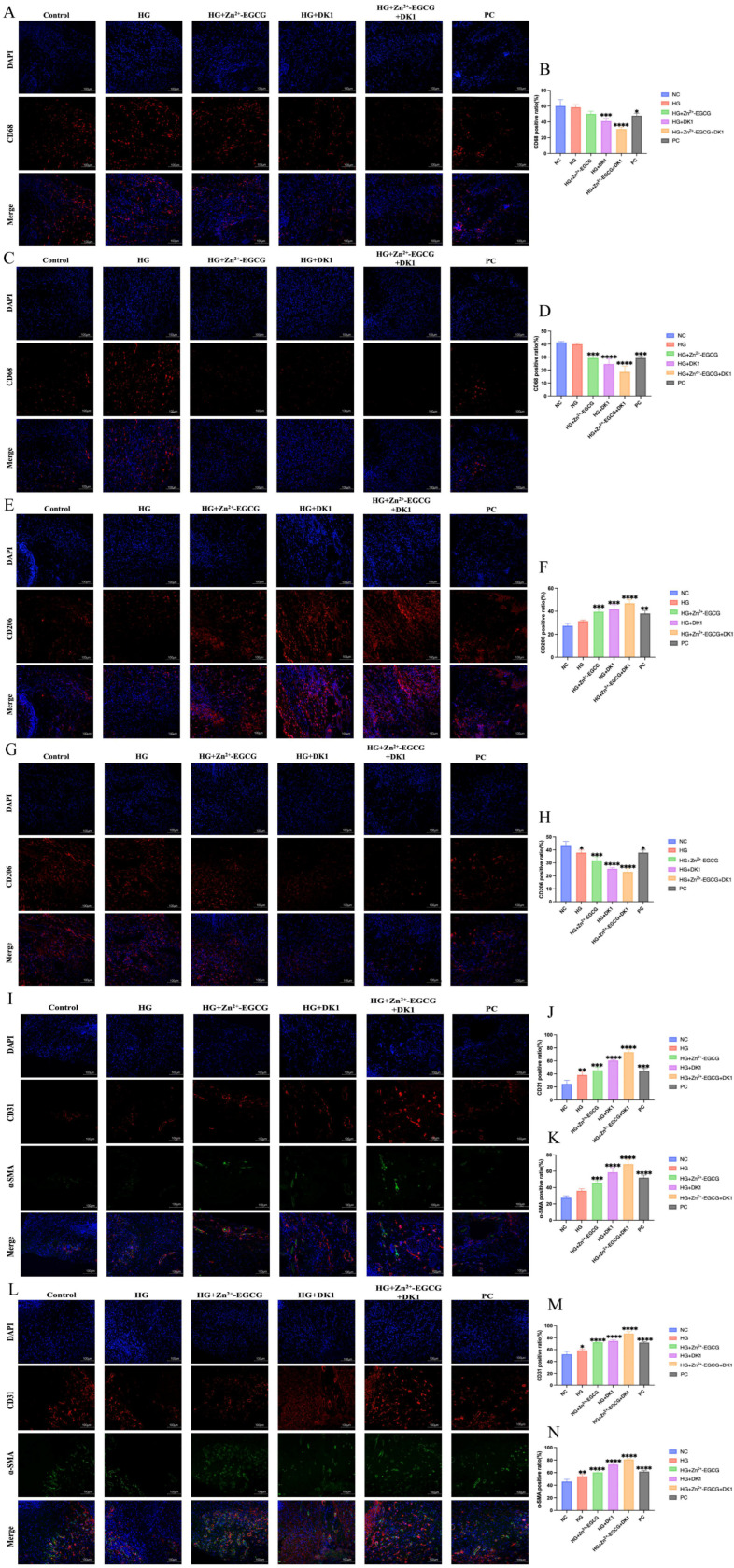
Immunofluorescence staining of wound inflammatory response and angiogenesis with DK1 hydrogel. (**A**) Immunofluorescence staining image of CD68 expression at the wound site on postoperative day 3, scale bar 100 μm. (**B**) Quantification of CD68 expression at the wound site on postoperative day 3. (**C**) Immunofluorescence staining image of CD68 expression at the wound site on postoperative day 7. (**D**) Quantification of CD68 expression at the wound site on day 7 post-surgery. (**E**) Immunofluorescence staining image of CD206 expression at the wound site on day 3 post-surgery, (**F**) Quantification of CD206 expression at the wound site on postoperative day 3. (**G**) Immunofluorescence staining image of CD206 expression at the wound site on postoperative day 7. (**H**) Quantification of CD206 expression at the wound site on postoperative day 7. (**I**) Immunofluorescence staining images of CD31 and α-SMA expression at the wound site on postoperative day 3. (**J**) Quantification of CD31 expression at the wound site on postoperative day 3. (**K**) Quantification of α-SMA expression at the wound site on postoperative day 3. (**L**) Immunofluorescence staining images of CD31 and α-SMA expression at the wound site on postoperative day 7. (**M**) Quantification of CD31 expression at the wound site on day 7 post-surgery. (**N**) Quantification of α-SMA expression at the wound site on day 7 post-surgery. Compared to the NC group, A *p*-value < 0.05 was considered statistically significant: (*) for *p* < 0.05, (**) for *p* < 0.01, (***) for *p* < 0.001, (****) for *p* < 0.0001.

Macrophages play a crucial role in regulating the inflammatory response during wound healing. CD68 is a marker for macrophages, and CD206 is a marker for anti-inflammatory M2 macrophages. We assessed the impact of DK1 hydrogel on inflammation by performing immunofluorescence staining for CD68 and CD206. The results showed that at days 3 and 7, the HG + Zn^2+^-EGCG + DK1 group had the lowest CD68 expression, indicating minimal inflammation (Figure 9A–D). CD206 expression was significantly upregulated on day 3, suggesting that this group effectively induced M2 macrophage polarization, reducing inflammation and promoting healing. By day 7, CD206 expression had rapidly decreased, indicating that the inflammation in this group had almost resolved (Figure 9E–H), providing strong evidence that DK1 hydrogel effectively regulates macrophage polarization to the M2 phenotype, further promoting wound healing. CD31 is a specific marker for endothelial cell differentiation, and α-SMA is a specific marker for smooth muscle cell differentiation. We evaluated the effect of DK1 hydrogel on angiogenesis at days 3 and 7 by immunofluorescence staining for α-SMA and CD31. The images showed that the HG + Zn^2+^-EGCG + DK1 group had a significantly greater number of new and mature blood vessels compared to the negative control group (Figure 9I–K). Quantitative analysis revealed that the HG + Zn^2+^-EGCG + DK1 group had the highest density of new and mature blood vessels (Figure 9L–N), indicating that DK1 hydrogel significantly enhanced angiogenesis during wound healing.

## 4. Discussion

Fibronectin is a key component of the extracellular matrix (ECM), and its biological function is highly dependent on the synergistic action of specific domains [38,39,40,42]. Different domains of fibronectin play distinct roles in biological activities. The diversity of functional domains allows fibronectin to bind to multiple partners such as collagen [43,44], fibrinogen [43,45], heparin [43,46], various cell receptors [43,47], and fibronectin itself [48]. This complex structure allows fibronectin to fulfill various biological roles, making it complex and multifaceted in wound healing [41]. Some studies have shown that fibronectin containing the D910 domains exhibits enhanced cell migration and adhesion activities [49,50]. Furthermore, the 8th, 9th and 10th domains, and the IIICS domain are confirmed to be binding sites for integrins [39]. The IIICS domain not only contains a heparin-binding site but also forms various splice variants, which are crucial for fibronectin’s function [39]. However, the functional studies on fibronectin containing these domains, particularly the combination of the IIICS domain with other domains, remain insufficiently explored.

In this study, three recombinant fibronectins with different domain combinations were successfully constructed: D89 (containing the 8th and 9th domains), D8910 (containing the 8th, 9th, and 10th domains), and DK1 (which introduces the IIICS domain into the D8910 construct). The results showed that all three recombinant fibronectins were expressed in a soluble form in *E. coli* expression systems. Furthermore, we evaluated the in vitro activities of the three recombinant fibronectins. The results revealed that although D89, D8910, and DK1 could all promote NIH/3T3 cell proliferation to some extent, the differences in cell adhesion and migration were particularly notable. In cell adhesion experiments, DK1 showed significantly higher adhesion efficiency than D89 and D8910 across various concentration gradients, reaching the highest adhesion rate at 40 µg/mL. This result suggests that the introduction of the IIICS domain likely enhances the interaction between DK1 and integrins or heparan sulfate proteoglycans on the cell surface, thus improving its initial adhesion ability. Some studies have suggested that the IIICS domain serves as a binding platform for various cell surface receptors, playing an important role in regulating cell spreading and adhesion stability [51,52,53]. Our findings further support this hypothesis on a functional level. Additionally, the IIICS domain is reported to be closely related to angiogenesis, as it may enhance the binding of fibronectin to integrins α5β1 or αvβ3 on endothelial cells, thereby activating downstream signaling pathways like FAK and PI3K/Akt, which promote cell migration and vascular reconstruction [54,55]. In HUVEC migration assays, DK1 also exhibited the most significant migratory effect. At early (6 h), middle (12 h), and late (24 h) time points, the wound healing rates of the DK1-treated group were significantly higher than those of the control, D89, and D8910 groups, with particularly notable advantages at higher concentrations. In contrast, while D89 and D8910 also promoted endothelial cell migration, their effects were less potent and sustained than DK1. These results suggest that the introduction of the IIICS domain not only enhanced DK1’s adhesion regulation on fibroblasts but also significantly improved its regulatory effect on endothelial cell migration. Since keratinocytes and endothelial cells respectively dominate epidermal regeneration and vascular remodeling, their functional coordination is key to successful wound healing [56,57,58,59], and thus, we conducted cell adhesion experiments. The results showed that DK1 also exhibited stable and significant adhesion-promoting effects on HaCaT and HUVECs, with a good dose-dependent relationship, indicating that DK1 has a strong advantage in promoting wound healing. This advantage is likely due to the synergistic effect between the IIICS domain and the D9–10 domains, making DK1 more similar in function to the physiological state of natural fibronectin.

Chronic diabetic wounds remain a major challenge in current clinical practice. Although preventive education and management strategies are crucial for reducing the incidence of diabetic foot ulcers [60], once an ulcer develops, conventional dressings or single growth factors often fall short in simultaneously addressing multiple obstacles such as inflammation regulation, angiogenesis, and tissue remodeling. Therefore, the development of bioactive materials with multifunctional regulatory capabilities has emerged as a key direction in diabetic wound healing research. In this study, based on DK1’s excellent in vitro bioactivity, we further combined it with a composite hydrogel system with good biocompatibility. Since Zn^2+^ and EGCG have good antibacterial and anti-inflammatory properties, they have been considered ideal components for addition to hydrogel dressings in recent years [61,62,63,64,65]. Therefore, we introduced Zn^2+^ and EGCG into the hydrogel system and systematically evaluated its repair effects in a diabetic mouse wound model. The results showed that the DK1 hydrogel significantly accelerated the wound healing process in diabetic mice at multiple time points, and at most time points, it was superior to the positive control dressing. This indicates that the DK1 hydrogel not only accelerates early wound contraction but also continuously promotes tissue regeneration during the middle and later stages, demonstrating stable and lasting healing effects. Notably, in the high-risk diabetic model, the DK1 hydrogel did not cause obvious ulceration or abnormal inflammatory reactions, further proving its good in vivo safety. Furthermore, immunohistochemistry analysis provided more direct evidence for the wound-healing effects of DK1 hydrogel. Specifically, the DK1 hydrogel group showed faster wound healing, gradual restoration of epidermal and dermal structures, and significant reduction in inflammatory cell infiltration. Compared to traditional dressings, DK1 hydrogel demonstrated stronger advantages in promoting collagen deposition and skin appendage regeneration, which was highly consistent with its in vitro ability to promote cell migration and proliferation. At the same time, immunological results further confirmed the immunoregulatory effects of DK1 hydrogel. In the early stage of wound healing, DK1 hydrogel significantly promoted M2 macrophage polarization, reducing the proportion of M1 macrophages, indicating its important role in reducing local inflammation and promoting tissue repair. This suggests that DK1 may promote the transition of the wound to the proliferation and remodeling stages through immune cell polarization. In addition, angiogenesis is a significant bottleneck in diabetic wound healing. Studies have shown that the IIICS region of fibronectin can enhance angiogenesis by promoting endothelial cell migration and improving microcirculation, which facilitates the delivery of oxygen and nutrients to the wound [66]. In this study, the DK1 hydrogel group also showed significant advantages in angiogenesis, reflected in a significant increase in new blood vessel density. This effect may be attributed to the IIICS domain’s promotion of endothelial cell function.

Although this study provides compelling evidence for the therapeutic potential of the DK1-functionalized hydrogel, several limitations should be acknowledged. First, a detailed characterization of the release profile of DK1 from the hydrogel matrix, as well as whether the presence of Zn^2+^ and EGCG influences DK1 release or its stability within the hydrogel, was not performed. Based on the chemical properties of these components, EGCG may form hydrogen bonds with DK1, thereby delaying its diffusion, while Zn^2+^ may coordinate with amino acid residues to stabilize the protein. The superior performance of the HG + Zn^2+^-EGCG + DK1 group in both in vitro and in vivo experiments indirectly suggests that any interactions among these components did not compromise DK1 bioactivity and may even contribute to synergistic effects; however, systematic release kinetics and protein stability studies are required to confirm this hypothesis. Second, although we observed enhanced M2 macrophage polarization and angiogenesis, the precise molecular signaling pathways involved—particularly the specific integrin activation downstream of the DK1 IIICS domain—require further mechanistic investigation. Addressing these limitations in future work will further enhance the clinical translation potential of this promising therapeutic strategy.

## 5. Conclusions

In this study, three recombinant fibronectin variants—D89, D8910, and DK1—were successfully constructed, and a DK1-functionalized hydrogel with favorable biocompatibility was developed. Among these variants, DK1 exhibited superior capabilities in promoting cell proliferation, adhesion, and migration in vitro. In a diabetic mouse wound model, the DK1-loaded hydrogel significantly accelerated wound closure, enhanced collagen deposition and remodeling, reduced the inflammatory response, promoted angiogenesis, and induced macrophage polarization toward the pro-regenerative M2 phenotype. This study highlights the critical role of the IIICS domain within DK1 in enhancing cellular interactions and immune modulation. Furthermore, the integration of Zn^2+^ and EGCG conferred antioxidant and antibacterial properties to the hydrogel while maintaining biocompatibility. This multifunctional platform represents a promising biomolecular therapeutic strategy for chronic diabetic wounds, holding significant potential for clinical application.

## Figures and Tables

**Table 1:** PCR amplification primer sequences for D89, D8910 and DK1.

Primer Name	Primer Sequence (5′~3′)
F-D89	TAACATATGGCGGTGCCGCCACCGAC
R-D89	TTAGGATCCTTAATCGCTCACGGTGCTCTGCTGACC
F-D8910	ATTCATATGGCGGTGCCGCCACCGAC
R-D8910	AATGGATCCTTAGGTGCGATAGTTAATGCTAATCGGT TTGCTGCT
F-DK1	AATTCCATATGGCGGTTCCGCCGCCGA
R-DK1	CGTCGAAGCTTTTACACATTCGGCGGG

## Data Availability

The datasets used and analyzed during the current study are available from the corresponding author upon reasonable request.

## Supplementary Materials

The following supporting information can be downloaded at: https://www.mdpi.com/article/10.3390/biom16040525/s1, Figure S1: Characterization of Hydrogel Physical Properties. Figure S2: Optimization of Expression Conditions for D89 Engineered Bacteria. Figure S3: Optimization of Expression Conditions for D8910 Engineered Bacteria. Figure S4: Optimization of Expression Conditions for DK1 Engineered Bacteria. Figure S5: Quantification of NIH/3T3 cell proliferation and adhesion rates for three recombinant fibronectins. Figure S6: Quantification of HUVEC cell migration rates for three recombinant fibronectins. Table S1: Bacterial endotoxin test results.
