# Supplementary material for: Hydrogel-Delivered Recombinant Fibronectin DK1 Promotes Diabetic Wound Healing by Boosting Cellular Responses
Source: Biomolecules. 2026 Apr 1;16(4):525. doi: 10.3390/biom16040525 (PMC13114212; doi:10.3390/biom16040525)
Supplement: Supplementary file 1 [file biomolecules-16-00525-s001.zip › Supplementary materials.pdf]

Supplementary materials

Figure

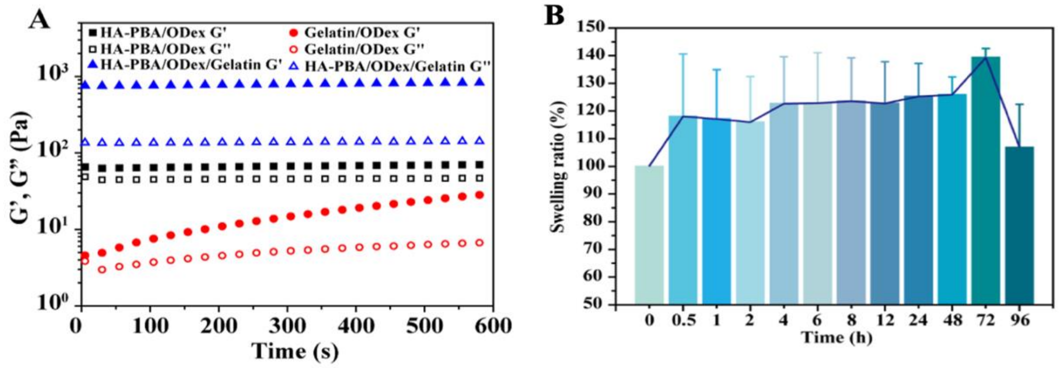

Figure S1 Characterization of Hydrogel Physical Properties. (A) Rheological Properties of the Hydrogel (B) Swelling Properties of the Hydrogel

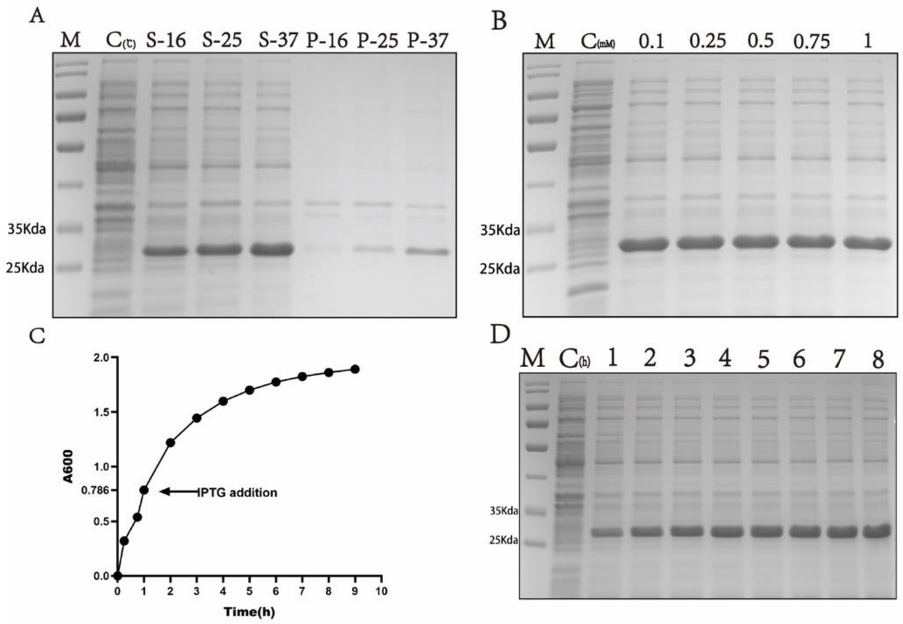

Figure S2 Optimization of Expression Conditions for D89 Engineered Bacteria. (A) Temperature Optimization for D89 Engineered Bacteria. M: Protein marker; C: Uninduced negative control; S-16: Supernatant from 16°C induction; S-25: Supernatant from 25°C induction; S-37: Supernatant from 37°C induction; P-16: Pellet from 16°C induction; P-25: 25°C induced pellet; P-37: 37°C induced pellet; (B) IPTG concentration optimization for D89 engineered bacteria; 0.1: 0.1 mM IPTG;

0.25: 0.25 mM IPTG; 0.5: 0.5 mM IPTG; 0.75: 0.75 mM IPTG; 1: 1 mM IPTG. (C): Growth curve of D89 engineered strain; (D): Induction time optimization for D89 engineered strain; M: Protein marker; C: Uninduced negative control; 1: Induced for 1h; 2: Induced for 2h; 3: Induced for 3h; 4: Induced for 4h; 5: Induced for 5h; 6: Induced for 6h; 7: Induced for 7h; 8: Induced for 8h;

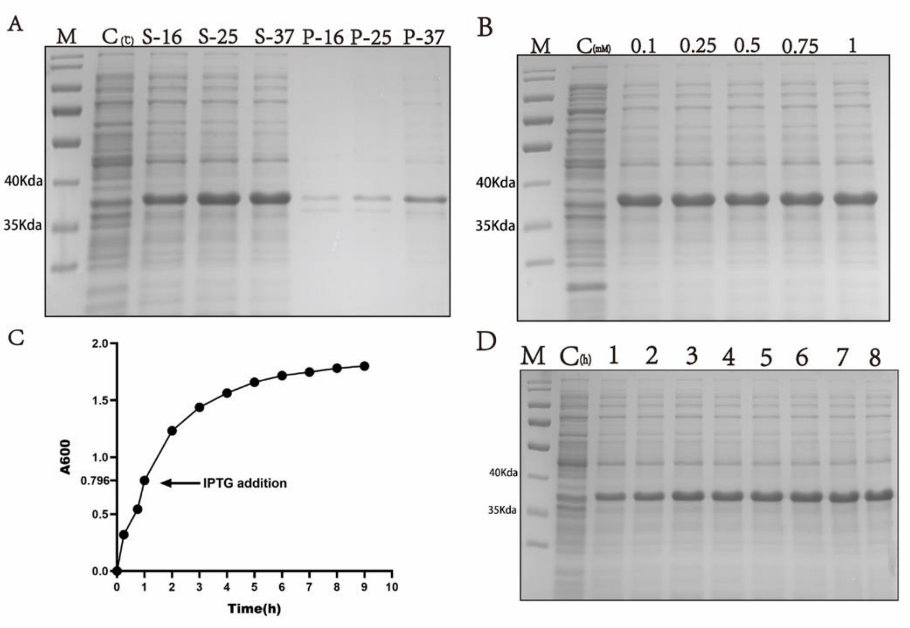

Figure S3 Optimization of Expression Conditions for D8910 Engineered Bacteria. (A) Temperature Optimization for D8910 Engineered Bacteria. M: Protein marker; C: Uninduced negative control; S-16: Supernatant from 16°C induction; S-25: Supernatant from 25°C induction; S-37: Supernatant from 37°C induction; P-16: Pellet from 16°C induction; P-25: 25°C induced pellet; P-37: 37°C induced pellet; (B) IPTG concentration optimization for D8910 engineered bacteria; 0.1: 0.1 mM IPTG; 0.25: 0.25 mM IPTG; 0.5: 0.5 mM IPTG; 0.75: 0.75 mM IPTG; 1: 1 mM IPTG. (C): Growth curve of D8910 engineered strain; (D): Induction time optimization for D8910 engineered strain; M: Protein marker; C: Uninduced negative control; 1: Induced for 1h; 2: Induced for 2h; 3: Induced for 3h; 4: Induced for 4h; 5: Induced for 5h; 6: Induced for 6h; 7: Induced for 7h; 8: Induced for 8h;

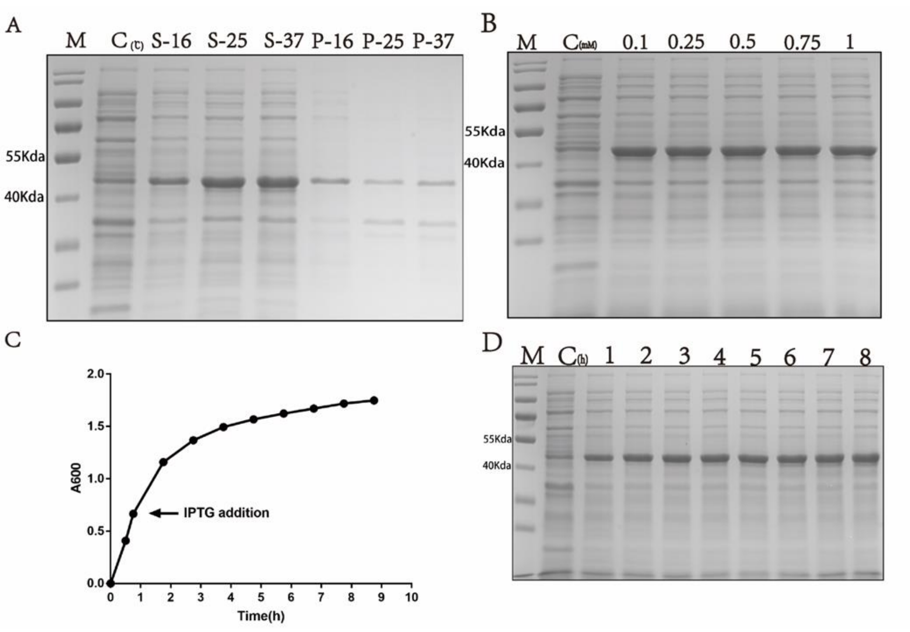

Figure S4 Optimization of Expression Conditions for DK1 Engineered Bacteria. (A) Temperature Optimization for DK1 Engineered Bacteria. M: Protein marker; C: Uninduced negative control; S-16: Supernatant from 16°C induction; S-25: Supernatant from 25°C induction; S-37: Supernatant from 37°C induction; P-16: Pellet from 16°C induction; P-25: 25°C induced pellet; P-37: 37°C induced pellet; (B) IPTG concentration optimization for DK1 engineered bacteria; 0.1: 0.1 mM IPTG; 0.25: 0.25 mM IPTG; 0.5: 0.5 mM IPTG; 0.75: 0.75 mM IPTG; 1: 1 mM IPTG. (C): Growth curve of DK1 engineered strain; (D): Induction time optimization for DK1 engineered strain; M: Protein marker; C: Uninduced negative control; 1: Induced for 1h; 2: Induced for 2h; 3: Induced for 3h; 4: Induced for 4h; 5: Induced for 5h; 6: Induced for 6h; 7: Induced for 7h; 8: Induced for 8h;

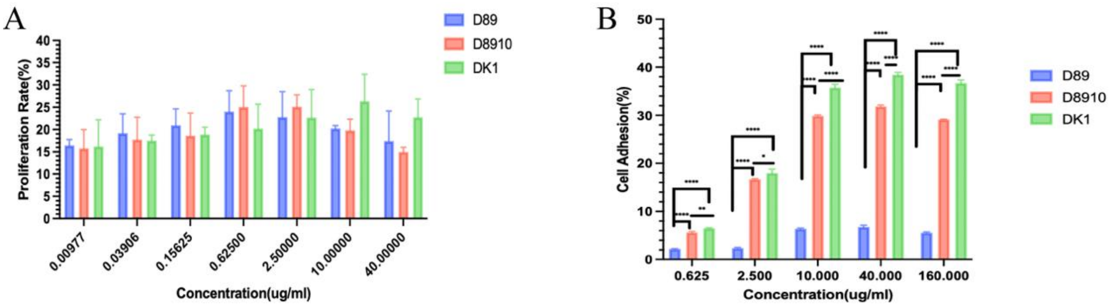

Figure S5 Quantification of NIH/3T3 cell proliferation and adhesion rates for three recombinant fibronectins. (A) Comparison of NIH/3T3 cell proliferation rates among the three recombinant fibronectins. (B) Comparison of NIH/3T3 cell adhesion rates induced by three recombinant fibronectin proteins.

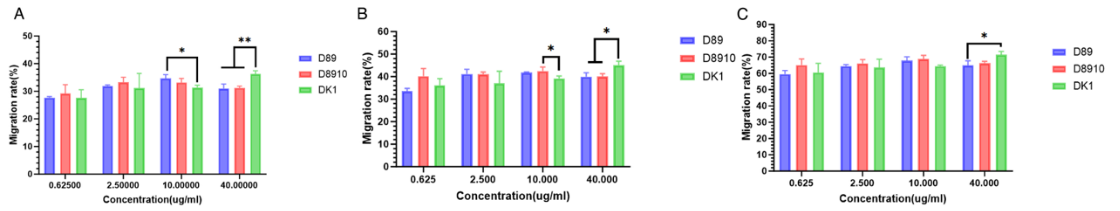

Figure S6 Quantification of HUVEC cell migration rates for three recombinant fibronectins. (A) Quantitative comparison of HUVEC cell migration rates at 6 hours for different concentrations of D89, D8910, and DK1. (B) Quantitative comparison of HUVEC cell migration rates after 12 hours at different concentrations of D89, D8910, and DK1. (C) Quantitative comparison of HUVEC cell migration rates after 24 hours at different concentrations of D89, D8910, and DK1.

Table

Table S1

Table S1 Bacterial endotoxin test results

Sample pretreatment: Dilute the sample solution 4 times as the test solution				
Sample	Test items	Test results	Test Unit	Detectin method
D89				
D8910	Bacterial Endotoxins	<0.5	EU/ml	Pharmacopoeia of the People's Republic of China, 2020 Edition, Part IV, General Chapter 1143, Method 1, Gel Limit Test
DK1				
